# Bismuth Tungstate Nanoplates—Vis Responsive Photocatalyst for Water Oxidation

**DOI:** 10.3390/nano13172438

**Published:** 2023-08-28

**Authors:** Tamer M. Khedr, Said M. El-Sheikh, Ewa Kowalska

**Affiliations:** 1 Institute for Catalysis, Hokkaido University, N21, W10, Sapporo 001-0021, Japan; 2 Nanomaterials and Nanotechnology Department, Central Metallurgical Research and Development Institute (CMRDI), P.O. Box 87 Helwan, Cairo 11421, Egypt; selsheikh2001@gmail.com; 3 Faculty of Chemistry, Jagiellonian University, Gronostajowa 2, 30-387 Krakow, Poland

**Keywords:** aurivillius phase perovskite, bismuth tungstate, nanoplates, mesoporous materials, O_2_ generation

## Abstract

The development of visible-light-responsive (VLR) semiconductor materials for effective water oxidation is significant for a sustainable and better future. Among various candidates, bismuth tungstate (Bi_2_WO_6_; BWO) has attracted extensive attention because of many advantages, including efficient light-absorption ability, appropriate redox properties (for O_2_ generation), adjustable morphology, low cost, and profitable chemical and optical characteristics. Accordingly, a facile solvothermal method has been proposed in this study to synthesize two-dimensional (2D) BWO nanoplates after considering the optimal preparation conditions (solvothermal reaction time: 10–40 h). To find the key factors of photocatalytic performance, various methods and techniques were used for samples’ characterization, including XRD, FE-SEM, STEM, TEM, HRTEM, BET-specific surface area measurements, UV/vis DRS, and PL spectroscopy, and photocatalytic activity was examined for water oxidation under UV and/or visible-light (vis) irradiation. Famous commercial photocatalyst–P25 was used as a reference sample. It was found that BWO crystals grew anisotropically along the {001} basal plane to form nanoplates, and all properties were controlled simultaneously by tuning the synthesis time. Interestingly, the most active sample (under both UV and vis), prepared during the 30 h solvothermal reaction at 433 K (BWO–30), was characterized by the smallest specific surface area and the largest crystals. Accordingly, it is proposed that improved crystallinity (which hindered charge carriers’ recombination, as confirmed by PL), efficient photoabsorption (using the smallest bandgap), and 2D mesoporous structure are responsible for the best photocatalytic performance of the BWO–30 sample. This report shows for the first time that 2D mesoporous BWO nanoplates might be successfully prepared through a facile template-free solvothermal approach. All the above-mentioned advantages suggest that nanostructured BWO is a prospective candidate for photocatalytic applications under natural solar irradiation.

## 1. Introduction

Environmental technology has received socioeconomic and scientific attention because of the global crises in energy, environment, water, and climate. Accordingly, photocatalytic water splitting (especially under natural solar radiation) has been suggested as a promising, sustainable, and eco-friendly “green” technology for hydrogen fuel production [1,2,3,4]. Regrettably, photocatalytic water oxidation (i.e., primary and key half-reaction of water splitting) is a major challenge because of the high energy barrier and the difficulty in both thermodynamics and kinetics [1,2,3]. Therefore, the development of novel photocatalysts for effective water oxidation is of high importance, and numerous studies focused on the development of effective photocatalysts, such as WO_3_ [5,6], BiVO_4_ [7,8], BiFeO_3_ [2], and Fe_2_O_3_ [9,10], have already been performed. For example, Djatoubai et al. prepared Ti–doped Bi_2_FeO_3_ nanoplates (with oxygen vacancies) by using a simple hydrothermal method that was followed by annealing in a hydrogen atmosphere, and created efficient photocatalytic water oxidation under vis irradiation [2]. It was proposed that improved activity could be attributed to the modulation of the electronic structure of Bi_2_FeO_3_ by incorporating titanium (doping) and forming oxygen vacancies, which resulted in enhanced light harvesting ability and charge carriers’ separation.

Bismuth tungstate (Bi_2_WO_6_; BWO), as one of the outstanding Aurivillius oxides, has received significant attention because of its tuned structures and compositions, as well as its outstanding performance, which makes it a promising VLR photocatalyst for different applications, specifically water oxidation [11,12]. Generally, russellite BWO (Aurivillius oxide with an orthorhombic structure) is built up by alternating (Bi_2_O_2_)^2+^ slabs and perovskite-like (WO_4_^2−^) layers [12]. The layered structure is advantageous for boosting the electron conductivity and improving the vis-harvesting ability (bandgap of ca. 2.6–2.9 eV) [11,12]. Kudo et al. were the first to prepared BWO for oxygen evolution reaction [13], and since their work, significant interest has been dedicated to the synthesis, estimation of property-controlled activities, mechanism investigation, improvement of photocatalytic performance, and various potential applications of BWO photocatalysts. It should be pointed out that BWO is considered to be a significant candidate for water oxidation (i.e., oxygen generation) because of its redox properties (valence band position), cost-effectiveness, nontoxicity, and adjustable morphology [11,12]. However, its further applications are extremely restricted because of its inherent limitations, such as poor capability to absorb visible light and speedy recombination of charge carriers. To remove these drawbacks, various BWO modification strategies have already been proposed, such as the elemental doping [14,15,16,17,18,19,20,21], surface modification (e.g., with noble metals [19,22,23,24,25] and carbon-based materials [26,27,28,29,30,31,32]), the structural optimization [23,27,33,34,35,36,37,38,39,40], and the construction of heterojunctions with other materials, such as Bi_2_WO_6_/TiO_2_ [41], Bi_2_WO_6_/g-C_3_N_4_ [42], Bi_2_WO_6_/MoS_2_ [43], Bi_2_WO_6_/FeS_2_ [44], Bi_2_WO_6_/CoIn_2_S_4_ [45], Bi_2_WO_6_/AgIO_3_ [46], Bi_2_WO_6_/CNT/TiO_2_ [47], Bi_2_WO_6_/g–C_3_N_4_/TiO_2_ [48], and Bi_2_WO_6_/BiOI/g–C_3_N_4_ [49].

It is well recognized that one- and two-dimensional nanomaterials might efficiently improve photocatalytic performance because of their distinct structure and appealing characteristics, promoting the charge carriers’ transfer [11,50,51]. Accordingly, 2D nanostructures (nanosheets/nanoplates) of BWO have also been synthesized, e.g., by the cetyltrimethylammonium bromide (CTAB)-assisted solvo/hydrothermal method [35,52,53,54,55,56,57,58,59,60]. However, CTAB (organic surfactant) is expensive, and the removal of its residues requires organic solvents like chloroform. According to our knowledge, a template-free synthesis of 2D BWO photocatalysts has not been reported yet. Therefore, this is the first paper in which the facile template-free solvothermal route (at different durations) is proposed for the preparation of 2D mesoporous BWO nanoplates.

## 2. Materials and Methods

### 2.1. Materials

Bismuth(III) nitrate pentahydrate (Bi(NO_3_)_3_·5H_2_O, 99.9%), sodium tungstate(VI) dihydrate (Na_2_WO_4_·2H_2_O, 99.9%), acetic acid (CH_3_COOH, 99%), ethanol (EtOH, 99.5%), and silver fluoride (AgF, 99%) were purchased from Wako Pure Chemical Co., Ltd. (Osaka, Japan). No further purification was performed on any of the chemicals. All experiments were conducted with ultrapure water (UP–H_2_O) obtained from a Direct–Q Millipore system.

### 2.2. Synthesis

Two-dimensional mesoporous BWO nanoplates were prepared using a facile template-free solvothermal method, as follows: Bi(NO_3_)_3_·5H_2_O (0.002 mol) and Na_2_WO_4_·2H_2_O (0.001 mol) were separately dispersed in 40 mL of acetic acid and UP–H_2_O, respectively. Subsequently, Na_2_WO_4_·2H_2_O solution was slowly dropped to Bi(NO_3_)_3_·5H_2_O solution under continuous agitation, stirred for 2 h, and then sonicated for 30 min. The final mixture was placed into a 100–mL Teflon-lined stainless-steel autoclave and then heated at 433 K for different durations (10, 15, 20, 25, 30, and 40 h). After cooling down, the obtained suspensions were centrifuged. The resultant solid samples were washed four times with UP–H_2_O and EtOH and finally dried overnight at 333 K. The obtained samples were abbreviated according to the duration of the solvothermal reaction, e.g., BWO–10 indicates the BWO sample prepared during 10 h synthesis.

### 2.3. Characterization of Photocatalyst

The physicochemical characteristics of synthesized BWO photocatalysts were investigated via different methods, as described further below. The crystalline compositions were examined by X-ray powder diffraction (XRD with accelerating voltage: 40 kV, emission current: 30 mA; Rigaku intelligent XRD SmartLab with a Cu target, Rigaku, LTD., Tokyo, Japan). Samples were analyzed between 10° and 90° at 1°/min scan speed and scan step of 0.0081°. The crystal structure parameters were determined with Rigaku PDXL software (Version 2.6.1.2, Rigaku, LTD., Tokyo, Japan, 2007–2015). To determine the crystallinity of BWO samples, an internal standard method was applied using commercial NiO as a standard. The standard with the crystallinity of 96.6% was thoroughly mixed in an agate mortar with BWO powder (20/80 weight ratio of NiO to BWO), and the resultant mixture was analyzed with an XRD diffractometer; then, the crystallinity of BWO samples was calculated.

The morphology was characterized by field emission-scanning electron microscopy (FE–SEM) under a high vacuum (JSM–7400F, JEOL, Tokyo, Japan). The images were captured in a wide range of magnifications in secondary electron imaging mode (SEI). Furthermore, scanning transmission electron microscopy (STEM; HD–2000, Hitachi, Tokyo, Japan) was also conducted in three different modes: secondary electron image (SE), Z contrast image (ZC), and phase contrast image (TE). Additionally, transmission electron microscopy (TEM) with an accelerating voltage of up to 200 kV, magnification power of up to 600 kX and resolution power down to 0.2 nm (JEOL–JEM 1230, Tokyo, Japan), high-resolution TEM (HR-TEM), and selected area (electron) diffraction were also applied for the characterization of the most active sample. The specific surface area (SSA) and the pore size (PS) distribution were estimated by N_2_ adsorption-desorption isotherms at 77 K employing Brunauer–Emmett–Teller (BET), and Brunauer–Joyner–Hallenda (BJH) analysis, respectively (Quanta Chrome Instruments, NOVA 2000 series, Peterborough, UK).

The photoabsorption characteristics were investigated on a diffuse reflectance spectroscope (DRS; JASCO V-670) equipped with a PIN-757 integrating sphere (JASCO, LTD., Pfungstadt, Germany) and using BaSO_4_ as a reference. The diffuse reflectance mode (R) was transformed into the Kubelka–Munk function F(R) to distinguish light absorption from scattering. The energy gap (Eg) values were estimated by plotting the (F(R) hv)^0.5^) versus the light energy (hv), where F(R) × E^0.5^ = ((1 − R)^2^/2R × hv)^0.5^ [60]. The photoluminescence (PL) emission spectra were recorded with a Shimadzu spectrofluorophotometer (RF-5301PC; λ_ex_ = 420 nm).

### 2.4. Photocatalytic Activity Tests

The photocatalytic water oxidation was carried out under UV and/or vis irradiation in the presence of in situ deposited silver as an electron scavenger. Typically, 0.05 g of BWO sample was dispersed in 5 mL of an aqueous suspension of AgF (0.05 mol L^−1^) in a 35 mL Pyrex test tube. The suspension (photocatalyst and AgF) was sonicated for ca. 10 min, deaerated with argon, and then the testing tube was sealed with a rubber septum and exposed to UV/vis (Hg lamp, λ > 290 nm) or vis (450 W–Xe lamp, λ > 400 nm; water IR filter, a cold mirror, and a cut–off filter: Y–42) irradiation. During the reaction, the suspension was constantly agitated in a thermostated water bath to keep the reaction temperature constant (about 298 K). The generated oxygen was quantified by employing a Shimadzu GC–8A chromatograph (Shimadzu Corporation, Kyoto, Japan), equipped with a thermal conductivity detector (TCD) and Poratak Q column (Agilent Technologies, Santa Clara, CA, USA). The recyclability tests were also performed (five repetitions) for the most active photocatalyst.

## 3. Results

### 3.1. Characterization

**Figure 1:**
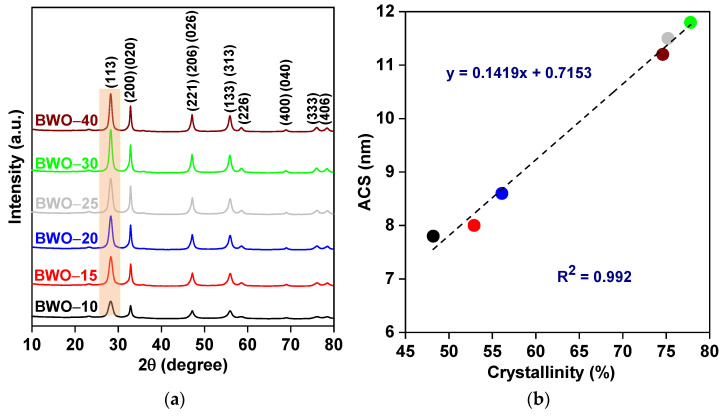
(**a**) XRD patterns; and (**b**) the correlation between crystallinity and average crystallite size (ACS) of BWO samples.

The successful fabrication of BWO was confirmed by XRD analysis, and the obtained XRD patterns are shown in Figure 1a. The characteristic peaks at 2–theta of ca. 28.4°, 32.8°, 47.4°, 55.8°, 58.8°, 68.7°, 75.9°, and 78.5° could be assigned to (113), ((200) (020)), ((221) (206) (026)), ((313) (133)), (226), ((400) (040)), (333), and ((406) (046)) planes of BWO, respectively. These crystal planes are characteristic for the orthorhombic morphology of BWO (which resembles a perovskite-like structure with lattice parameters of *a* = 5.456 Å, *b* = 5.436 Å, and *c* = 16.428 Å, card no. 66579), and are consistent with previous findings [14,38,61,62,63]. Moreover, the lack of other diffraction peaks indicates the high crystal purity of the prepared samples. Although the duration of the solvothermal reaction shows no effect on the position of diffraction peaks, the increase in peaks’ intensity and peaks’ sharpening with the prolongation of reaction duration indicates the crystal growth and the improvement of crystallinity. Indeed, the crystallinity of BWO, estimated by an internal standard method, is improved by prolonging the reaction duration up to 30 h (Table 1). However, a further increase in the reaction time (40 h) causes a slight decrease in the crystallinity, which could be caused by the higher density of crystalline defects. Therefore, it might be concluded that the best crystalline properties are achieved during 30 h treatment (BWO–30), i.e., longer and shorter solvothermal time results in the formation of an imperfect crystalline structure. Similarly, Zhang et al. have also confirmed the influence of reaction duration (hydrothermal process) on the crystallinity of BWO [33].

Additionally, it has been found that the ratio of I_(113)_/I_(200)_ (intensity of respective peaks) is lower than the standard value of 5, implying an anisotropic growth along the {001} basal plane, which suggests the formation of square-plate morphology (2D), as discussed later. It has already been suggested that large crystallites could be formed by an increase in solvo/hydrothermal time (as observed by intense and sharp XRD peaks) [33,62,64]. Accordingly, the average crystallite size (ACS) has been estimated, using the full-width half-maximum (FWHM) of (113) diffraction peak with the Debye–Scherrer equation [38], and obtained data are listed in Table 1. The ACS increases from 7.8 to 11.8 nm with an increase in the reaction duration till 30 h, and then slightly decreases to 11.2 nm for 40 h time. Therefore, it might be proposed that crystal growth is associated with crystallinity, as confirmed in Figure 1b. Accordingly, it has been found that both crystallinity and crystallite size are mainly controlled by the duration of the solvothermal reaction. Similarly, Li and coworkers found a correlation between the conditions of hydrothermal reaction and crystalline properties of BWO samples, i.e., an increase in crystallite sizes from 13.6 nm to 16.2 nm as the temperature of the hydrothermal reaction increased from 413 K to 433 K [25].

Summarizing, it might be concluded that the duration of the solvothermal reaction has a significant impact on the crystalline properties of BWO photocatalysts. Moreover, the estimation of optimal conditions for photocatalysts’ preparation is crucial for the preparation of perfect crystals. Similar findings have already been found for both BWO [33,62,65,66] and other photocatalysts, such as TiO_2_ [67,68,69,70,71], ZnO [72], MoS_2_ [73,74], and g–C_3_N_4_ [75].

**Figure 2:**
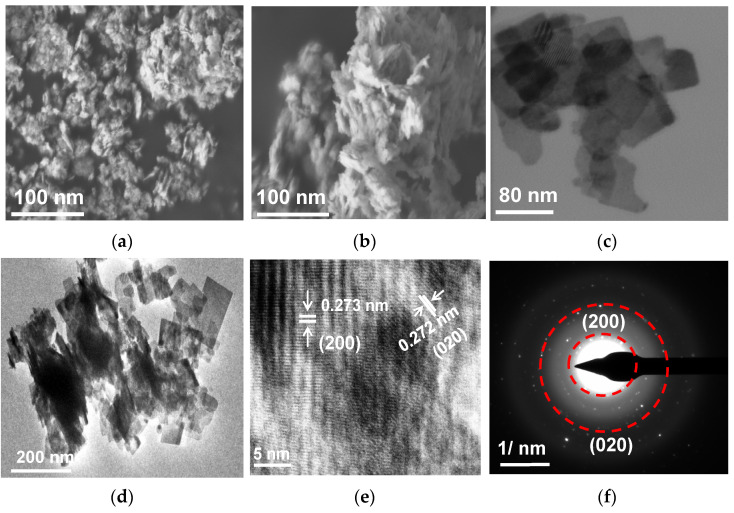
(**a**) FE-SEM of BWO–10 sample; (**b**) FE-SEM, (**c**) STEM, (**d**) TEM, (**e**) HRTEM, and (**f**) SAED images of BWO–30 sample.

The microscopic observations (Figure 2) have confirmed the self-assembly of nanoplate subunits during solvothermal growth, resulting in the formation of a layered microstructure. The obvious difference between BWO–10 and BWO–30 (a and b images, respectively) indicates that the small nanoparticles tend to aggregate and coalesce (forming larger particles with a clear–cut appearance), which is consistent with the XRD results (Table 1). The 2D structure of the BWO–30 sample is also clearly seen in STEM (Figure 2c) and TEM (Figure 2d) images. It should be noted that the BWO–30 sample consists of uniformly dispersed square nanoplates with smooth and transparent surfaces. To elucidate the growth orientation of the nanoplates, an HRTEM observation with SAED analysis was also carried out, and the obtained observations are displayed in Figure 2e,f. Accordingly, two sets of lattice fringes could be clearly observed, as seen by a marked interplanar spacing of ca. 0.273 nm and 0.272 nm, indexed to the (200) and (020) planes, respectively, of orthorhombic BWO (Figure 2e). Moreover, the SAED pattern exhibits a regular diffraction spot array with a spacing of 0.273 and 0.272 nm, corresponding to the (200) and (020) planes, respectively (Figure 2f). Therefore, it might be concluded that 2D BWO nanoplates preferably grow along the {001} facets, which is consistent with XRD results. This might be caused by a higher atom density on the {001} facets, leading to a slower growth along the {001} of BWO nanoplates [33,53,54,55,76,77].

**Figure 3:**
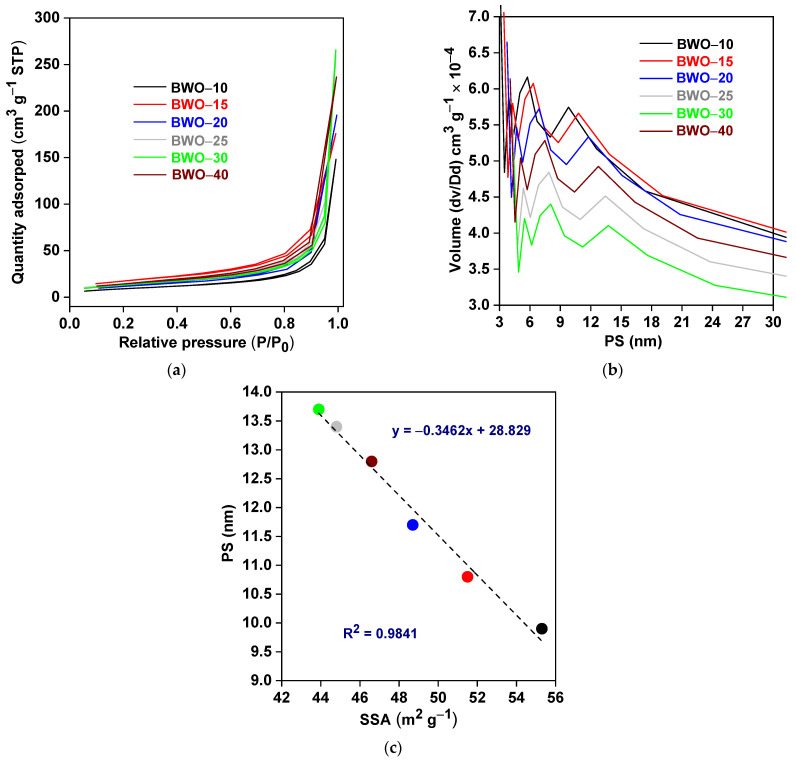
(**a**) N_2_ adsorption-desorption isotherms; (**b**) pore size (PS) distribution; and (**c**) correlation between the specific surface area (SSA) and PS of the BWO samples.

To investigate the textural properties, BET/BJH measurements were performed at 77 K, and the obtained results are shown in Figure 3 and Table 2. All samples exhibit similar isotherms, categorized as type IV (according to IUPAC), with small hysteresis loops with a relative pressure of ca. 0.7–0.9, as shown in Figure 3a. This might be caused by irregular voids, resulting from large particles’ (plates) packing. Meanwhile, the surface characteristics were further estimated, and the obtained results, including the specific surface area (SSA), pore volume (PV), and pore size (PS), are presented in Figure 3b and Table 2. It has been found that the largest values of SSA (55.3 m^2^ g^−1^) and PV (5.8–6.2 × 10^−4^ cm^3^ g^−1^) are obtained for the BWO–10 sample, prepared during the shortest reaction (10 h). Clearly, the SSA and PV values decrease with increasing the reaction duration up to 30 h (43.9 m^2^ g^−1^ and 4.1–4.4 × 10^−4^ cm^3^ g^−1^, respectively). All obtained data correlate well with ACS, i.e., an increase in ACS (prolonged reaction) corresponds to a decrease in SSA. The longer reaction time might significantly affect the crystal growth since it allows smaller grains to grow, forming bigger crystallites. These large nanocrystallites might also be pushed into the channels of mesopores, decreasing the pore volume. It is clearly observed that all samples exhibit a mesoporous structure (PS = 3.9–13.7 nm). Figure 3c shows a clear correlation between SSA and pore size.

**Figure 4:**
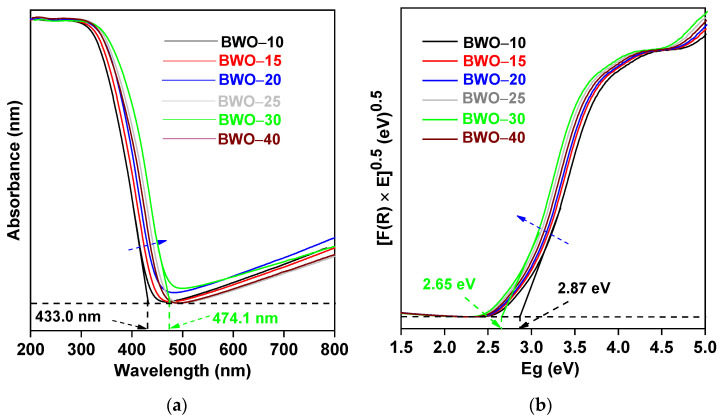
(**a**) UV-vis diffuse reflectance spectra; (**b**) the corresponding Kubelka–Munk reflectance spectra for BWO samples.

The photoabsorption features of BWO photocatalysts are displayed in Figure 4 and Table 2. Indeed, all photocatalysts could absorb a significant portion of visible light, i.e., the absorption edge (AE) ranges from 433.0 nm to 474.1 nm (Figure 4a). Therefore, prepared BWO samples are expected to act as VLR photocatalysts. Moreover, an obvious bathochromic shift in the AE (and the consequent bandgap narrowing, as displayed in Figure 4b) with an increase in the solvothermal time correlates well with an increase in particle/crystallite size (size-dependent light absorption), as the phonon frequency constant increases with an increase in crystal size [78,79,80,81]. The comparison of photoabsorption properties demonstrates that the absorption edge at the shortest wavelength (433 nm), and thus the sample with the largest energy bandgap (2.87 eV), was prepared during the shortest duration of solvothermal reaction (BWO–10), whereas BWO–30 exhibits the narrowest energy bandgap and the absorption edge at the longest wavelength (i.e., 2.65 eV and 474.1 nm, respectively), as presented in Figure 4 and Table 2.

### 3.2. Photocatalytic O_2_ Evolution

**Figure 5:**
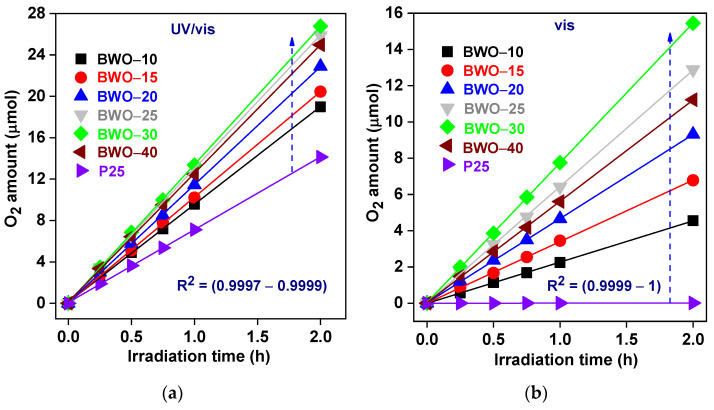
The photocatalytic O_2_ generation: (**a**) under UU/vis; and (**b**) vis irradiation over BW-10, BWO-15, BWO-20, BWO-25, BWO-30, BWO-40, and P-25 photocatalysts.

The photocatalytic activity of BWO samples in comparison to that by famous P25 (common and standard photocatalyst [82,83]) was evaluated through photocatalytic O_2_ generation in an aqueous solution under UV and/or vis illumination in the presence of in situ deposited silver as an electron scavenger. It was found that O_2_ gas was not generated in the absence of either a photocatalyst (direct photolysis) or light (under dark conditions). Hence, it might be concluded that water oxidation must proceed via a photocatalytic mechanism, i.e., the formation of charge carriers under irradiation. Indeed, O_2_ evolution was observed in the presence of photocatalysts (BWO and P25) upon UV/vis and vis irradiation, as presented in Figure 5 (and Table 3). A linear evolution of oxygen for all samples during the whole duration of the reaction confirms the photocatalytic mechanism (not a “light-initiated” dark reaction), also revealing the high photostability of all materials.

Under UV/vis irradiation, all BWO samples exhibit much higher activity than that of one of the most active titania photocatalysts (P25) with similar surface properties (BET of ca. 50 m^2^ g^−1^ [84]) (Figure 5a). However, the crystallinity of BWO samples is much worse than that in P25 (>90% estimated by the same method [84]), but ACS of BWO is smaller than that in titania (25.3 and 39.6 nm for anatase and rutile, respectively) [84]). Accordingly, it might be concluded that a well-organized nanostructure (2D) might be responsible for the better performance of BWO. Moreover, as expected, P25–TiO_2_ is hardly active under vis irradiation due to a much wider bandgap (>3.0 eV) and, thus, a shorter wavelength edge (λ < 400 nm) (Figure 5b). The photocatalytic activity of BWO photocatalysts enhances with an increase in the synthesis time up to 30 h, reaching 13.40 μmol h^−1^ and 7.74 μmol h^−1^ of oxygen evolution rate under UV/vis and vis, respectively (BWO–30 sample), as shown in Figure 5 and Table 3.

**Figure 6:**
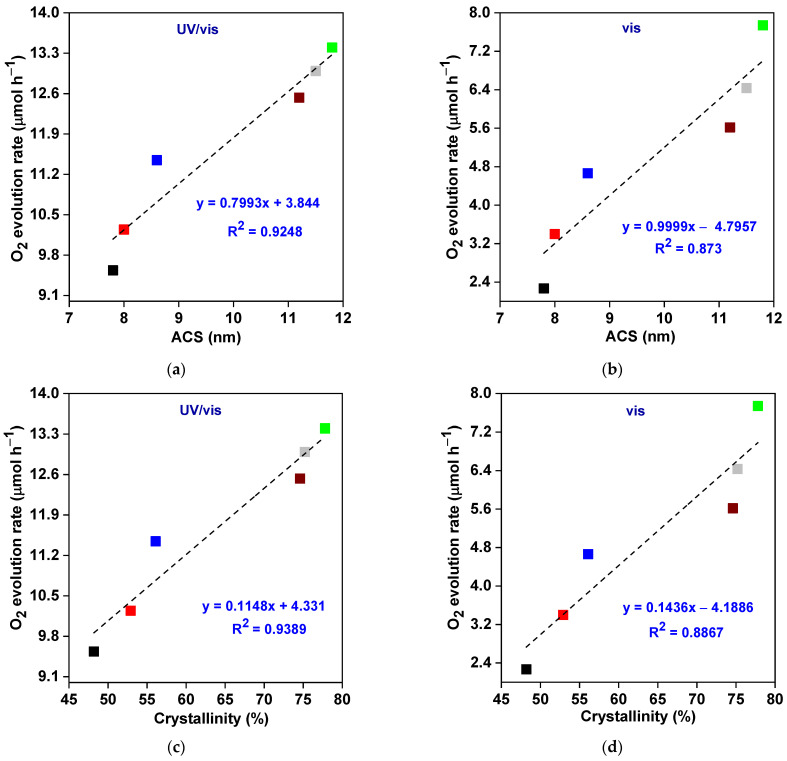
The correlation between photocatalytic activity and average crystallite size (ACS) (**a**,**b**); and crystallinity% (**c**,**d**) of BWO samples.

**Figure 7:**
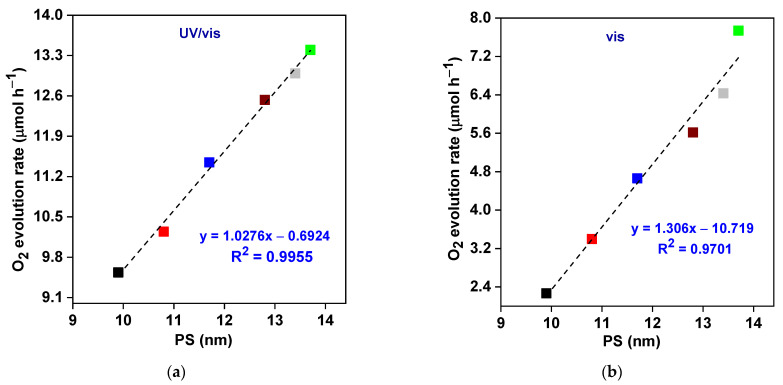
The correlation between photocatalytic activity and pore size (PS) of BWO samples: under (**a**) UV/vis; and (**b**)vis irradiation.

**Figure 8:**
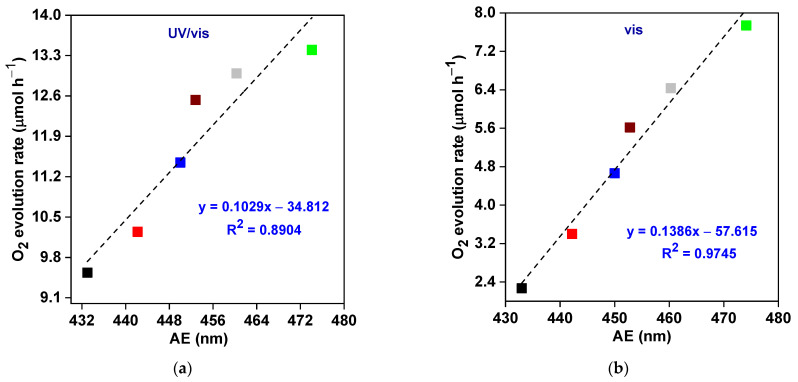

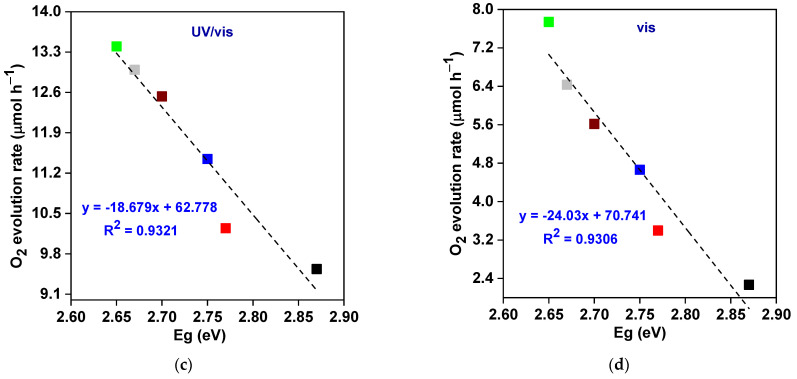
The correlation between photocatalytic O_2_ evolution (under UV/vis and vis irradiation) and (**a**,**b**) absorption edge (AE); and (**c**,**d**) bandgap energy (Eg) of BWO samples.

Notably, the specific surface area is commonly considered one of the main factors controlling photocatalyst performance (similar to “dark” catalytic reactions). In this context, a large specific surface area could: (i) enhance the incident light-harvesting ability, (ii) create surface-active sites, (iii) promote the reactant molecules’ adsorption on the surface of the photocatalyst, (iv) increase the rate of reactants’ formation (e.g., reactive oxygen species), and thus significantly boost the photocatalytic performance for various applications (specifically, photocatalytic degradation of hazardous organic compounds) [85,86,87,88]. Surprisingly, the most active sample (BWO–30) is characterized by the smallest specific surface area (Table 2), implying that the specific surface area of the fabricated photocatalysts is not the reason behind increasing the photocatalytic activity in the current study. Similar findings have also been observed elsewhere [89,90,91,92]. It might be proposed that, in the case of direct redox reactions via photogenerated charges (here, silver reduction and water oxidation by photo-formed electrons and holes, respectively), instead of the involvement of intermediates (such as reactive oxygen species formed on the surface of photocatalyst; crucial for oxidative decomposing of organic compounds), the specific surface area does not govern photocatalytic performance. Therefore, other properties should be decisive for photocatalytic performance. Among them, crystallinity, nanoplate morphology, mesoporous structure (PS = 5.5–13.7 nm), and improved light absorption ability (especially under vis) must be considered. Indeed, the photocatalytic activity of the fabricated BWO materials correlates well with other properties (Figure 6, Figure 7 and Figure 8), i.e., crystallite size, crystallinity, pore size, absorption edge, and energy bandgap.

Intriguingly, crystallite size and crystallinity are two of the most crucial factors that significantly impact the photocatalytic activity of semiconductor photocatalysts [93,94]. However, contrary results can be found in the literature regarding the influence of crystallite/particle size on photocatalytic performance, i.e., the positive [93,95,96] and negative [94,97,98,99] impacts. In this study, the reaction rate under both UV/vis and vis irradiation increases upon an increase in the crystallite size, and the BWO–30 sample with the largest crystallites also exhibits the best photocatalytic activity, as depicted in Figure 6a,b. These findings could be attributed to enhancing the optical characteristics and promoting the charge carriers’ separation and mobility [93]. Moreover, it has been pointed out that the optimal value of crystallite size lies in the range of 7–15 nm for diverse photocatalytic applications [93,100,101]. In contrast, there is no disagreement about the impact of crystallinity on photocatalytic activity, as it is well-documented that higher crystallinity means better photocatalytic performance. The improved crystallinity correlates with lower content of crystalline defects, and thus a lower rate of charge carriers’ recombination (as confirmed also here, by PL data; discussed further). Similarly, the dependence of crystallinity on charge carriers’ separation efficiency (and thus photocatalytic activity) has already been proven for other photocatalysts, e.g., BiFeO_3_ [81] and TiO_2_ [102]. Therefore, the BWO–30 sample possessing the largest crystallinity shows the best activity towards O_2_ evolution under UV/vis and vis irradiation (Figure 6c,d). Here, during the formation a of 2D structure, it might be proposed that the large size of BWO–30 indicates the perfectly formed crystal, and thus the largest size corresponds to the best crystallinity.

Remarkably, the mesoporous structure introduces efficient paths for reactants’ transfer to reactive sites (mass transfer) and also improves multiple scattering of light, resulting in effective photon absorption and, hence, boosting photocatalytic activity [103,104,105]. As displayed in Figure 7a,b, the photocatalytic O_2_ evolution rate (under UV/vis and vis irradiation) improves upon an increase in the pore size of the synthesized BWO specimen, and the BWO–30 sample with the largest pore size exhibits the best photocatalytic activity. This might be caused by improved water adsorption and enhanced light penetration into the photocatalyst in the presence of larger pores, as already suggested in other reports [103,104,105].

Moreover, photocatalytic activity firmly relies on the optical properties of photocatalysts. In this sense, the photocatalytic O_2_ generation rate (under UV/vis and vis irradiation) increases upon an increase in the absorption edges (the efficient use of more photons) and, thus, a decrease in energy bandgaps (Eg) of fabricated BWO (the BWO–30 sample shows the superior photocatalytic performance) (Figure 8a–d). In addition, the nanoplate morphology is beneficial for charge carriers’ separation/transfer and efficient light harvesting ability, allowing multiple irradiation reflection (similar to photonic crystals [106]). Therefore, it should be concluded that the photocatalytic activity of BWO might be significantly enhanced by changing the physical properties via reaching the optimal conditions of solvothermal reaction, e.g., reaction time (here: 30 h). However, the prolonged reaction might also cause a decrease in photocatalytic activity because of a reduction in the quality of materials. For example, a 40 h solvothermal reaction causes a decrease in crystallinity (a higher density of crystalline defect).

**Figure 9:**
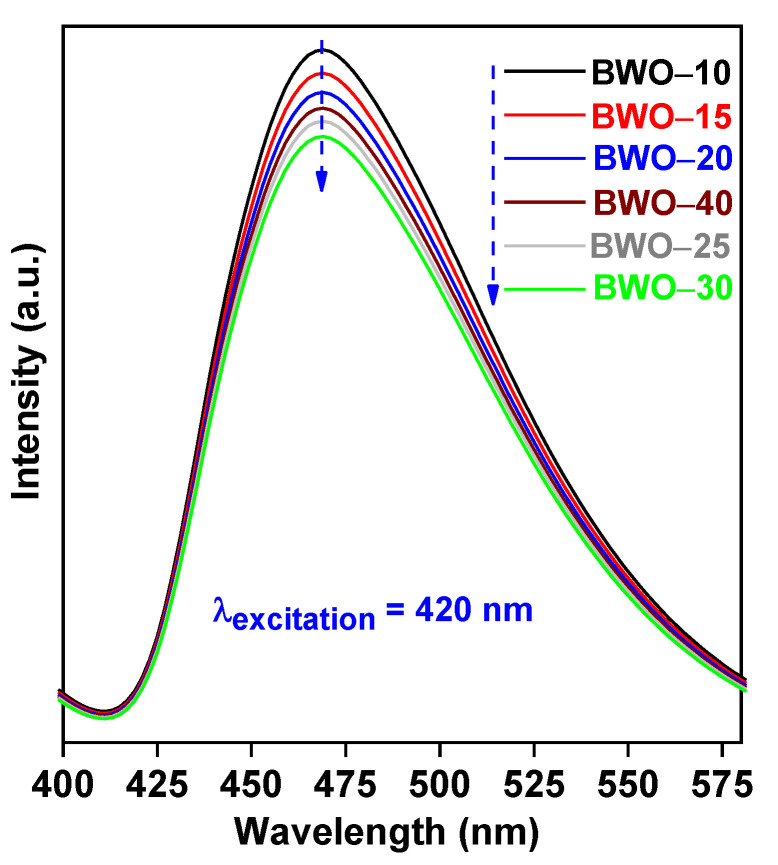
PL spectra of BWO samples.

To confirm that properties (mainly crystallinity) influence the h^+^/e^−^ recombination and, thus, the photocatalytic efficiency, PL spectroscopy was carried out, and the findings are presented in Figure 9. All BWO photocatalysts display photoluminescence at ca. 468.7 nm (after excitation at 420 nm), which correlates well with charge carries’ recombination [107]. However, the PL intensities between samples differ significantly. Indeed, the BW–30 sample with the highest crystallinity (and photocatalytic activities) possesses the weakest PL intensity (i.e., the best efficiency of photoinduced charge carriers’ separation), whereas the strongest PL signal (i.e., the fastest charge carries’ recombination rate) is obtained by the BWO–10 sample with the worst crystal properties. It has been confirmed that reaching optimal conditions of solvothermal reaction is important for getting the best photocatalytic performance, and thus, prolonged reaction (here > 30 h) might cause the preparation of samples with unfavorable crystalline defects.

**Figure 10:**
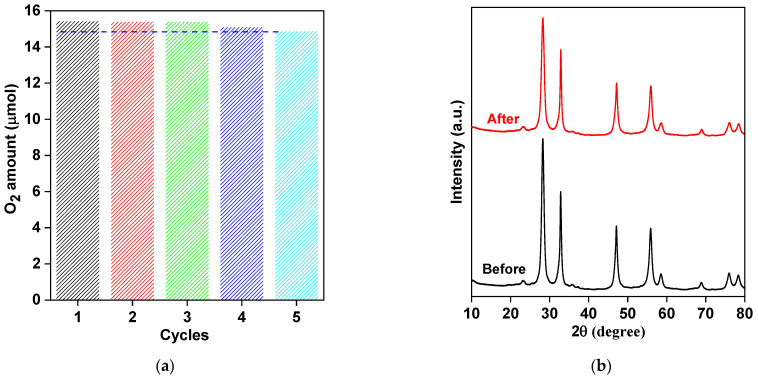
(**a**) Recyclability of the BW-30 in the photocatalytic O_2_ generation under vis irradiation; (**b**) XRD patterns of BWO-30 before and after the photocatalytic reaction.

To confirm the long-term photostability, the recyclability experiments were carried out for the most active sample (BW–30) during five cycles under vis irradiation, and the obtained data are displayed in Figure 10a. The high photocatalysis stability has been confirmed with only a slight change in oxygen generation between the first and fifth cycles (from 15.45 μmol to 14.89 μmol). Furthermore, XRD analysis of a recycled sample indicates that crystalline properties have not been changed (Figure 10b). Therefore, it could be proposed that 2D mesoporous BWO nanoplates are promising photocatalysts for vis applications, especially oxidation reactions). Indeed, 2D BWO photocatalysts exhibit superior photocatalytic activity toward O_2_ evolution, as compared to recently reported photocatalytic materials (Table 4) [2,108,109,110].

**Figure 11:**
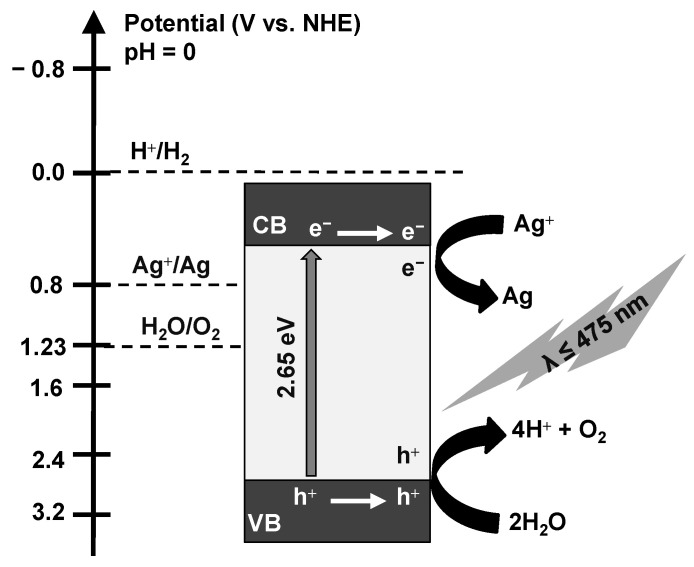
Schematic diagram of the proposed mechanism of photocatalytic O_2_ generation over BWO (BWO-30) photocatalyst.

Finally, the mechanism of photocatalytic water oxidation on BWO photocatalyst (BWO–30) might be proposed (Figure 11). The edge potentials of the valence band (E_VB_) and conduction band (E_CB_) have been estimated using the Mulliken electronegativity theory (Equations (1) and (2)) [111]:

E_CB_ = χ − E^e^ − 0.5E_g_ (1)

E_VB_ = E_CB_ + E_g_ (2)

Here, E_g_ is the energy bandgap of BWO–30 (2.65 eV); χ is the absolute electronegativity of Bi_2_WO_6_ (6.36 eV); and E^e^ is free electrons energy in the hydrogen scale (4.5 eV). Accordingly, estimated E_CB_ and E_VB_ are equal to 0.54 and 3.19 V, respectively. Therefore, it is possible to reduce silver cation (Ag^+^) into silver atom (Ag), as the E_CB_ (0.54 V) of the photocatalyst is less positive than the standard redox potential of Ag^+^/Ag (0.7996 V). Moreover, water can be oxidized into O_2_, as the E_VB_ (3.19 V) of the photocatalyst is more positive than the standard redox potential of H_2_O/O_2_ (1.23 V). Based on the above results, the mechanism of photocatalytic water oxidation over BW–30 (in the presence of AgF as an electron scavenger) has been proposed as the following: The h^+^/e^−^ pairs are generated under irradiation (both UV and vis could be used) since electrons (e^–^) are transferred from the VB to the CB, leaving holes (h^+^) at VB of the photocatalyst (Equation (3)).

The photogenerated charge carriers (e^−^_CB_ and h^+^_VB_) migrate to the photocatalyst surface. Finally, the Ag^+^ cation (from AgF in the reaction suspension, Equation (4)) reacts with e^−^_CB_ to produce zero-valent silver (Equation (5)) and h^+^_VB_ oxidizes water to form O_2_ (Equation (5)). Furthermore, the obtained Ag nanoparticles, loaded on the surface of BWO, might improve the photocatalytic activity and performance (working as a co-catalyst). It should be pointed out that the released proton (from water oxidation, Equation (6)) could not be reduced into H_2_, because the E_CB_ of BWO–30 photocatalyst (0.54 V) is more positive than the standard redox potential of H^+^/H_2_ (0.0 V). Therefore, acidification of reaction suspension is proposed, e.g., as summarized in exemplary Equation (7).

Bi_2_WO_6_ + hν → e^−^_CB_ + h^+^_VB_ (3)

AgF → Ag^+^ + F^−^ (4)

e^−^_CB_ + Ag^+^ → Ag (5)

2h^+^_VB_ + H_2_O → 2H^+^ + ½ O_2_ (6)

H^+^ + F^−^ → HF (7)

## 4. Conclusions

The facile template-free solvothermal reaction has proven to be an efficient method for the preparation of a 2D porous BWO photocatalyst. BWO material shows significant activity in photocatalytic water oxidation under both UV and vis irradiation. The conditions of the solvothermal reaction are critical for the properties of BWO and, thus, the resultant photocatalytic performance. It has been found that a 30 h solvothermal reaction at 433 K results in the preparation of the most active photocatalyst with the best properties, i.e., nanoplate morphology (2D), mesoporous structure, high crystallinity (low content of defects) and efficient light harvesting ability. To the best of our knowledge, this is the first work displaying that 2D mesoporous BWO nanoplates could be successfully synthesized by a template-free solvothermal method and subsequently used for enhanced photocatalytic water oxidation under UV/vis and vis irradiation. Therefore, this study opens the door for other applications of BWO photocatalysts under natural solar radiation.

## Figures and Tables

**Table 1:** The crystalline properties of BWO samples.

Sample ID	Hydrothermal Time (h)	^1^ d (113) (Ǻ)	^2^ FWHM (Ǻ)	^3^ ACS (nm)	Crystallinity (%)
BWO–10	10	3.162	1.0371	7.8	48.2
BWO–15	15	3.160	1.0104	8	52.9
BWO–20	20	3.159	0.9450	8.6	56.1
BWO–25	25	3.157	0.7149	11.5	75.2
BWO–30	30	3.156	0.6847	11.8	77.8
BWO–40	40	3.158	0.7191	11.2	74.6

^1^ d: d–spacing; ^2^ FWHM: full–width half–maximum; ^3^ ACS: average crystallite size.

**Table 2:** Textural and optical properties of BWO samples.

Catalyst ID	^1^ SSA/m^2^ g^−1^	^2^ PV/cm^3^ g^−1^ × 10^−4^	^3^ PS/nm	^4^ AE/nm	^5^ Eg/eV
BWO–10	55.3	5.8–6.2	3.9–9.9	433.0	2.87
BWO–15	51.5	5.7–6.0	4.3–10.8	442.2	2.77
BWO–20	48.7	5.3–5.7	4.7–11.7	450.0	2.75
BWO–25	44.8	4.5–4.8	5.4–13.4	460.3	2.67
BWO–30	43.9	4.1–4.4	5.5–13.7	474.1	2.65
BWO–40	46.6	4.9–5.4	5.1–12.8	452.8	2.70

^1^ SSA: specific surface area; ^2^ PV: pore volume; ^3^ PS: pore size; ^4^ AE: absorption edge; ^5^ Eg: energy bandgap.

**Table 3:** Photocatalytic activity over P25–TiO_2_ and BWO photocatalysts.

Catalyst ID	Under UV-Vis Irradiation			Under Vis Irradiation		
	Evolved O_2_ Amount/ μ mol	Evolved O_2_ Rate/ μ mol h^−1^	R^2^	Evolved O_2_ Amount/ μ mol	Evolved O_2_ Rate/ μ mol h^−1^	R^2^
BWO–10	18.99	9.54	0.9998	4.55	2.27	0.9999
BWO–15	20.45	10.24	0.9999	6.78	3.40	0.9999
BWO–20	22.89	11.44	0.9997	9.31	4.66	0.9999
BWO–25	25.87	13.00	0.9999	11.22	5.62	0.9999
BWO–30	26.78	13.40	0.9999	15.45	7.74	1.0000
BWO–40	24.98	12.53	0.9998	12.89	6.43	0.9999
P25	14.13	7.10	0.9998	0.011	0.006	0.9999

R^2^: regression coefficient.

**Table 4:** Comparison of photocatalytic O_2_ evolution efficiency over the prepared BWO photocatalyst and other photocatalysts in recent works.

Catalyst/ Dose (g)	Light Source	Reactant Suspension	Irradiation Time (min)	O_2_ Rate (μmol h^−1^)	Ref.
g-C_3_N_4_/ Ag_3_PO_4_/ 0.3	white LED light	100 mL aq. solution (1 g AgNO_3_)	60	3.30	[108]
g-C_3_N_4_/ MoS_2_ Ag_3_PO_4_/ 0.3	white LED light	100 mL aq. solution (1 g AgNO_3_)	40	6.99	[109]
Ti/ BiFeO_3_/ 0.01	300-W Xe lamp, λ > 420 nm; UV cut–off filter: Y–42)	80 mL aq. solution (0.14 g AgNO_3_ + 0.16 g La_2_O_3_)	360	2.74	[2]
MoS_2_/ MnWO_4_/ 0.05	300-W Xe lamp, λ > 420 nm; UV cut–off filter: Y–42)	200 mL aq. solution (0.03 M AgNO_3_ + 0.2 g La_2_O_3_)	180	5.19	[111]
2D Bi_2_WO_6_/ 0.05	Hg lamp, λ > 290 nm	5 mL aq. solution (0.05 M AgF)	120	13.40	This work
	300-W Xe lamp, λ > 420 nm; UV cut–off filter: Y–42)			7.74	

## Data Availability

The data presented in this study are available on request from corresponding author (T.M.K.).
